# Reliability Analysis of Concrete Beam with High-Strength Steel Reinforcement

**DOI:** 10.3390/ma15248999

**Published:** 2022-12-16

**Authors:** Feiyan Zhang, Fan Feng, Xiang Liu

**Affiliations:** 1 School of Management Engineering, Zhejiang Guangsha Vocational and Technical University of Construction, Dongyang 322100, China; 2 School of Architectural Engineering, Hunan Institute of Engineering, Xiangtan 411100, China; 3 School of Civil Engineering, Fujian University of Technology, Fuzhou 350118, China

**Keywords:** reliability analysis, high-strength steel reinforcement, flexural capacity, concrete beam

## Abstract

In concrete structures, replacing conventional steel bars with high-strength steel reinforcement (HSSR) can effectively save the use of materials. However, the deformation properties and strength dispersion of HSSR are different from those of conventional steel reinforcement, which restricts or conservatively uses them in practical applications. For example, the partial safety factor of HRB500 grade steel bars (the yield strength is 500 MPa) in guideline GB50010-2010 is larger than that of conventional steel bars, and there is no relevant guidance for HRB600 grade steel bars (the yield strength is 600 MPa). Based on this, this paper will propose the limit state design method of high-strength steel reinforced concrete beam (HSSRCB) based on reliability analysis, which is convenient for the popularization and use of HSSR. Firstly, the flexural performance test of HSSRCBs was introduced, and the flexural capacity of HSSRCB was analyzed based on the existing prediction model. Second, a sectional numerical analysis model was established, where the section was discretized into several points, and then the curvature was gradually increased to obtain the corresponding bending moment through integration. A large number of samples were calculated to obtain statistical characteristics of the error of prediction model. Then, the limit state functions were established for two kinds of format, including partial safety factor format (PSSF) and resistance reduction factor format (RRFF), respectively, and the reliability of HSSRCBs was analyzed based on Monte Carlo simulation. Finally, the recommended values of partial safety factor of material and reduction factor of bearing capacity were proposed, in which the design strength of HRB500 and HRB600 reinforcement was 454 MPa and 545 MPa for PSSF, respectively, and the resistance reduction factor for the flexural capacity of HSSRCB was 0.8 and 0.75 for RRFF, respectively.

## 1. Introduction

In the reinforcement concrete structure, the use of high-strength steel reinforcement (HSSR) instead of conventional steel reinforcement can effectively save materials when the performance reaches the standard. At present, steel reinforcement with yield strength exceeding 500 MPa are called HSSR [1,2].

HSSR is characterized with a limited ductility capacity and reduced energy dissipation. This is one of the major concerns of the application of HSSR [1]. In addition, the effective combination of HSSR and concrete materials is an important guarantee for the application of HSSR in engineering. Therefore, it is necessary to fully study the HSSR concrete structures. Shahrooz et al. [1] studied the ductility and crack control of flexural concrete members with HSSRs, and found that the strain limits for HSSR must be changed to achieve the curvature ductility comparable to that implicit with the current use of HSSR. Li and Aoude [3] investigated the effects of steel fiber on the static and blasting properties of beams made of high-strength concrete (HSC) and HSSR. Liao [4] studied the anti-explosion performance of HSSR concrete beams, and the influence of HSSR on the dynamic response and damage characteristics of reinforcement concrete beams was analyzed. Harries et al. [2] analyzed the bending crack width of RC beams with high-strength ASTM A1035 steel bars, and concluded the demonstrated conservativeness of existing ACI and AASHTO crack control provisions allows present specifications to be extended to the anticipated higher service level stresses associated with the use of HSSR. Zhao et al. [5] conducted shear tests on HSSR concrete beams, and revised the calculation of bearing capacity suggested in CECS38:2004 and ACI544.4R. Deng et al. [6] studied the fatigue performance of high-strength reinforced UHPC beams, and the results showed that the beams still exhibit stable structural performance after concrete cracking. Zhang et al. [7] investigated the shear behavior of ultra-high-performance concrete (UHPC)–normal concrete (NC) composite beams reinforced by HSSR, and the results showed that under the same shear span ratio, the shear cracking load and ultimate shear capacity of UHPC–NC composite beams are increased by 11.8–94.1% and 16–66.6%, respectively, compared with the NC beams. Various research results showed that the use of HSSR can effectively improve the mechanical properties of concrete structures, especially the bearing capacity.

The above literatures have analyzed the mechanical properties of concrete members with HSSR, which are of great significance for the application of HSSR. The test or practice results in the above literatures show that the bearing capacity of members with HSSR has certain variability, this is because there were many uncertainties in the material characteristics and construction errors of the structure, as well as in the loads (including dead load and live load) effect on the structure, and the prediction models also had errors. To ensure the safety of the structure, these uncertainties cannot be ignored. For this, reliability theory is usually used for analysis. Some scholars focus on reliability analysis theory, for examples, Zhao et al. [8] and Tong et al. [9] proposed a high-order moment method based on linear moment method. Zhang et al. [10] proposed an efficient method for time-variant reliability including finite element analysis. Chen and Yang [11] proposed a method called direct probability integral method; this method can calculate both static and dynamic reliability [12,13]. Another set of scholars studied the bearing capacity of concrete structures based on reliability theory; for example, Zhou et al. [14] analyzed the capacity of FRP shear strengthened reinforced concrete beams. Zhang et al. [15] analyzed the reliability of flexural capacity of concrete beams reinforced with hybrid BFRP and steel rebars. Wakjira et al. [16] used a variety of machine learning methods to predict the flexural bearing capacity of reinforced concrete beams strengthened with fabric-reinforced cementitious matrix. The results showed that the flexural bearing capacity of beams predicted by existing analysis models was very discrete. For this reason, the reliability analysis method was used to propose the resistance reduction factor of the prediction model. The same method was applied in shear-critical RC beams strengthened with inorganic composites [17]. Shen et al. [18] used Monte Carlo simulation and machine learning-based substitution model (ML-MCS) to calibrate the reliability of floor–column joints in actual engineering cases, analyzed the sensitivity of random variables, and deeply studied the impact of this analysis on structural reliability.

Although there have been many studies on the mechanical properties of concrete structure with HSSR, there are few studies on its reliability, and the limit state design method of conventional reinforced concrete members was usually directly applied in HSSR concrete member. Moreover, in the guideline GB50010-2010 [19], the design strength of HSSR is conservative, or even has no relevant provisions; for example, it is specified that the strength design value of HRB500 reinforcement (yield strength is 500 MPa) is 435 MPa, that is, the partial safety factor $\gamma_{y}$ is 1.15, and it is higher than the conventional steel reinforcement ($\gamma_{y}$ = 1.11), and there is no corresponding provision for HRB600 reinforcement. The concrete flexural member (beam) is a basic member of structure, in which the tensile longitudinal reinforcement plays an important role in the bearing capacity of bending. Based on the above reasons, this paper will take flexural beam as the research object, and investigate the reliability of bearing capacity of RC structures with HSSR, and propose corresponding material partial safety factors or resistance reduction factors, and these will provide a theoretical basis for the construction industry of HSSR.

## 2. Flexural Capacity Analysis of RC Beam with HSSR

### 2.1. Experimental Analysis

#### 2.1.1. Details of Test Specimens

Four high-strength steel reinforcement concrete beam (HSSRCB) specimens were designed and manufactured, with the section size of 250 mm × 400 mm, and the lengths of beam were all 2700 mm; the support spacing of the beam were 2400 mm. The thickness of concrete cover of each specimen was 25 mm; the upper longitudinal reinforcement of the concrete beam adopts two HPB300 reinforcements with a diameter of 10 mm as the erection reinforcement, and the other longitudinal reinforcements were set as the bottom reinforcement; the stirrups adopted the HRB400 steel reinforcement with a diameter of 10 mm, and the spacing was 80 mm. According to the guideline GB50010-2010 [19], shear failure of concrete beams with this reinforcement scheme can be avoided. More details can be found in Table 1.

**Figure 1:**
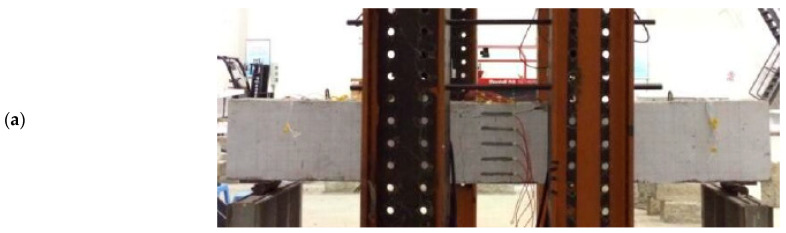

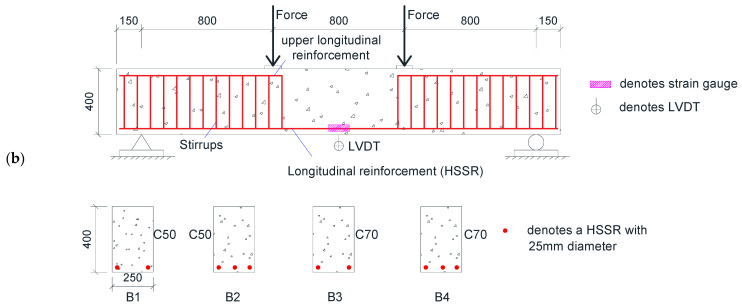
Loading diagram of specimens: (**a**) test photos; (**b**) geometry, midspan section, and reinforcement details (unit: mm).

The destructive test was carried out on the beam specimens in the form of four-point-loading, in which two actuators were used to simultaneously apply vertical load/displacement to the beam specimens, as shown in Figure 1. At the initial loading stage of the specimen, force control was adopted as the loading mode, and the loading rate was 1 kN/s. When the force reaches 70% of the estimated ultimate bearing capacity of the specimen, it was switched to displacement control, and the loading rate is 0.1 mm/s. When the load drops below 80% of the ultimate load, the specimen would be unloaded. In order to obtain the deformation of beam, some linear variable displacement transducers (LVDTs) were installed at the bottom of the beam [20].

#### 2.1.2. Materials

The mechanical properties of steel reinforcement are shown in Table 2.

The material mechanical properties of concrete were tested using a series of 150 mm cubes. According to GB50010-2010 [19], the axial compressive strength can be obtained by the following conversion method,

(1) $$f_{ck}=0.88\alpha_{c1}\alpha_{c2}f_{cu,k}$$

where $\alpha_{c1}$ is the ratio of compressive strength of prism to cube, and when the concrete strength grade is not greater than C50, $\alpha_{c1}$ = 0.76, and when the concrete strength grade is greater than C80, $\alpha_{c1}$= 0.82, and when the concrete strength grade is an intermediate value, interpolation is made between 0.76 and 0.82; $\alpha_{c2}$ denotes the concrete brittleness coefficient, and when the concrete strength grade is not greater than C40, $\alpha_{c2}$ = 1, and when the concrete strength grade is greater than C80, $\alpha_{c2}$ = 0.87, and interpolation is used between C40 and C80; $f_{cu,k}$ is the compression strength of the concrete cube.

The results of cube compression strength and axial compression strength are shown in Table 3.

#### 2.1.3. Experimental Results

**Figure 2:**
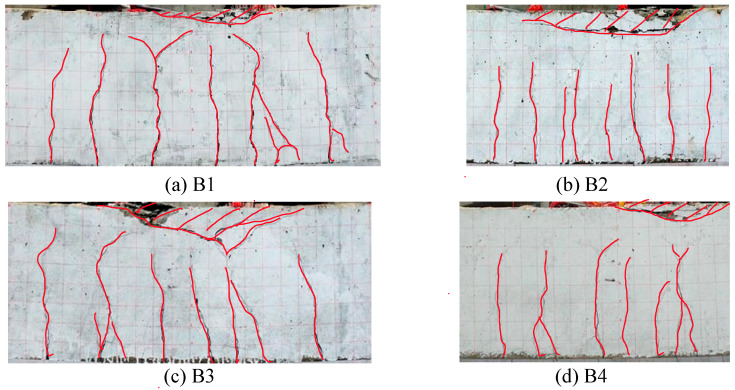
Failure mode of specimens.

During the test, the cracks were all concentrated in the pure bending section. At the initial stage of loading, cracks first appeared at the bottom of the mid-span of the beam. With the increased of load, the width and number of cracks also increased, and the directions of the cracks were nearly perpendicular to the beam longitudinal direction. After specimens yielded, the cracks rapidly expanded to the top of the beam, and the deformation increased significantly, until the upper concrete at the middle span was crushed, and each specimen presented typical bending failure, as shown in Figure 2.

**Figure 3:**
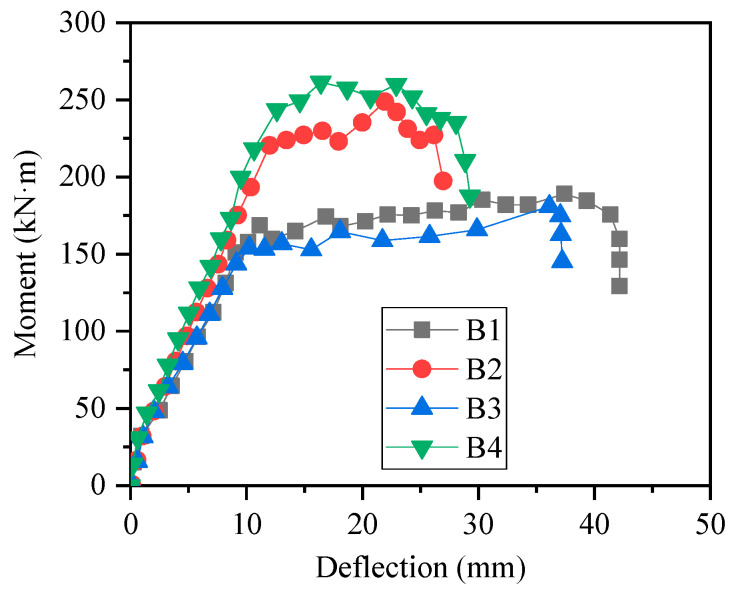
Moment–deflection curves of specimens.

The section moment–deflection curves of each test specimen are shown in Figure 3. It can be seen that at the initial stage of loading, the test specimen was in an elastic stage. After the bottom concrete cracked (about 25 kN), the slopes of the moment–deflection curves changed, which means that the stiffnesses of the test specimen changed. When the steel reinforcement yield, the load rose slowly or no longer rose.

The key points of results are summarized in Table 4. Comparing different specimens, it can be found that the concrete strength had little influence on the trend of moment–deflection curves and had little influence on the yield load and ultimate load of specimens. The reinforcement ratio had a significant impact on the trend of the moment–deflection curves, which has a greater influence on the yield load, ultimate load, and deformation performance. The yield load and ultimate load of specimens with large reinforcement ratio (B2 and B4) were greater than those with small reinforcement ratio (B1 and B3); this is because when the reinforcement ratio increases, the reinforcement involved in resisting bending moment increases, and the reinforcement ratio contributes greatly to the bending moment bearing capacity, which leads to the obvious increase of yield moment and ultimate moment; while the deformation performance of specimens with large reinforcement ratio (B2 and B4) was weaker than that of specimens with small reinforcement ratio (B1 and B3). This is because when the reinforcement ratio increased, the strain of the reinforcement under the same bending deformation decreased, and the crack developed slowly. The concrete in the compression area was crushed before the concrete in the tension area had completely cracked, which can also be seen in Figure 2b,d. In short, in the range of proper reinforced beams, with the increase of reinforcement ratio, the bending capacity beams will increase, while the ductility will decrease. In other words, in the case of the same reinforcement ratio, the use of HSSR can effectively improve the bending capacity of concrete beams, but its ductility will be reduced.

### 2.2. Numerical Model

**Figure 4:**
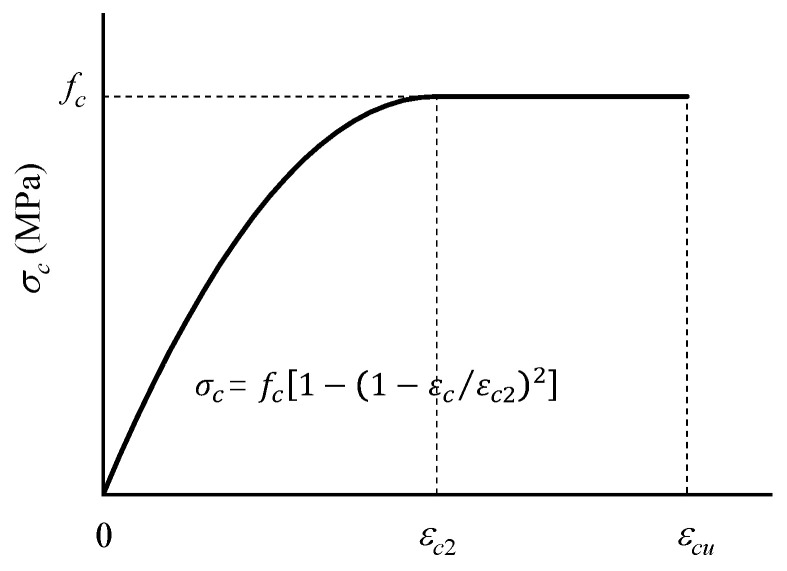
Compressive constitutive model of concrete.

To obtain enough samples of HSSRCB, it is necessary to use numerical calculation methods. The numerical model used was a kind of section analysis method. There are some assumptions: (1) The contribution of concrete tension was not considered; (2) the tensile constitutive model of reinforcement was bi-linear model; (3) the compressive constitutive model of concrete can be expressed as (see in Figure 4) [19,22],

(2) $$\sigma_{c}\left(\varepsilon_{c}\right)=\{\begin{array}{cc} 0, & \varepsilon_{c}<0\\ f_{c}\left[1-{\left(1-\frac{\varepsilon_{c}}{\varepsilon_{0}}\right)}^{2}\right], & 0\leq \varepsilon_{c}{<\varepsilon }_{0}\\ f_{c}, & \varepsilon_{c2}\leq \varepsilon_{c}{<\varepsilon }_{\mathrm{cu}}\\ 0, & \varepsilon_{c}{>\varepsilon }_{\mathrm{cu}} \end{array}$$

where $\varepsilon_{c}$ is the concrete strain, and $\sigma_{c}$ is the concrete stress; *f_c_* is the compressive strength; $\varepsilon_{0}$ is the compressive strain when the stress just reaches *f_c_*, and it equals to 0.002; $\varepsilon_{\mathrm{cu}}$ is the ultimate compressive strain, and it equals to 0.0035.

**Figure 5:**
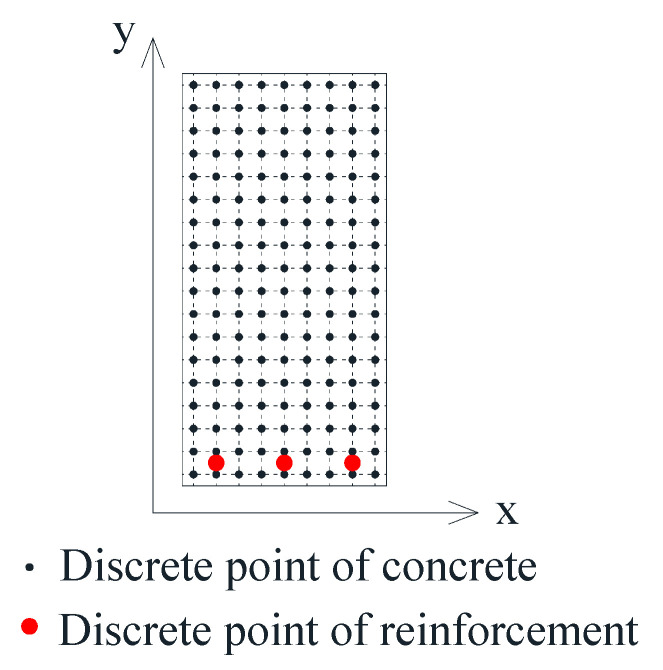
Section discretization.

The section was discretized into several points, as shown in Figure 5. According to the knowledge of material mechanics, the section bending moment can be obtained through the section quadrature, and it can be written as follow,

(3) $$M=\int_{\Omega }^{}\left(y_{c}-y\right)\sigma_{c}dxdy+\overset{n_{s}}{\underset{j=1}{\sum }}\left(y_{c}-y_{sj}\right)A_{si}\sigma_{si}$$

where Ω denotes section domain; $\sigma_{c}$ is the stress of concrete; $A_{si}$ and $\sigma_{si}$ denotes area and stress of *i*-th steel reinforcement; $y_{sj}$ is the location of *i*-th reinforcement; *n_s_* denotes the number of steel reinforcement.

By Gaussian quadrature, Equation (2) can be simplified as

(4) $$M=\frac{A}{n}\overset{n}{\underset{i=1}{\sum }}\left(y_{c}-y_{i}\right)\sigma_{c}\left(x_{i},y_{i}\right)+\overset{n_{s}}{\underset{j=1}{\sum }}\left(y_{c}-y_{sj}\right)A_{si}\sigma_{si}$$

where *A* is the section area, *n* is the total number of discrete points. $\sigma_{c}\left(x_{i},y_{i}\right)$ denotes the stress of *i*-th discrete point of concrete.

The bending moment values corresponding to different curvatures were obtained through the integration of points step by step. According to Fujikake et al. [23], the deformation can be calculated by the formula,

(5) $$\delta =\phi L^{2}/12$$

where ϕ is the curvature of section, L is the span of beam.

More details of the numerical model can be found in Melo et al. [22]. Since the numerical model was only based on a section analysis method, it was only applicable to normal section failure.

**Figure 6:**
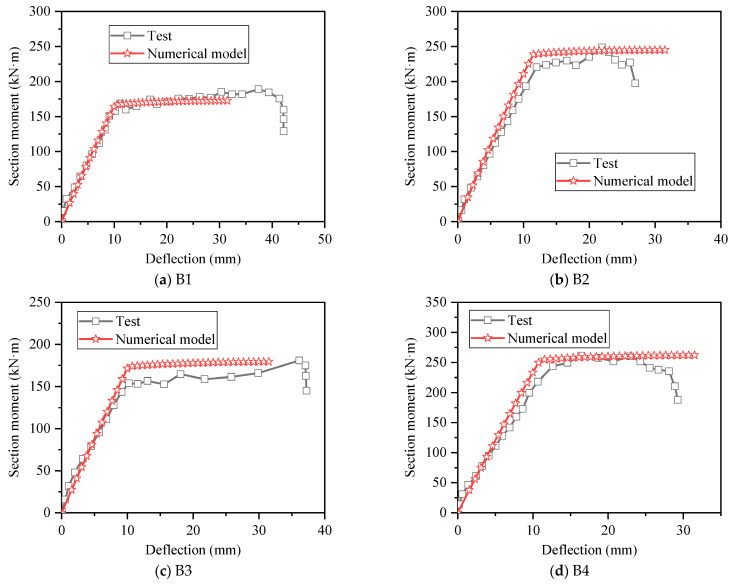
Comparison of moment–deflection curves between numerical and test results.

**Figure 7:**
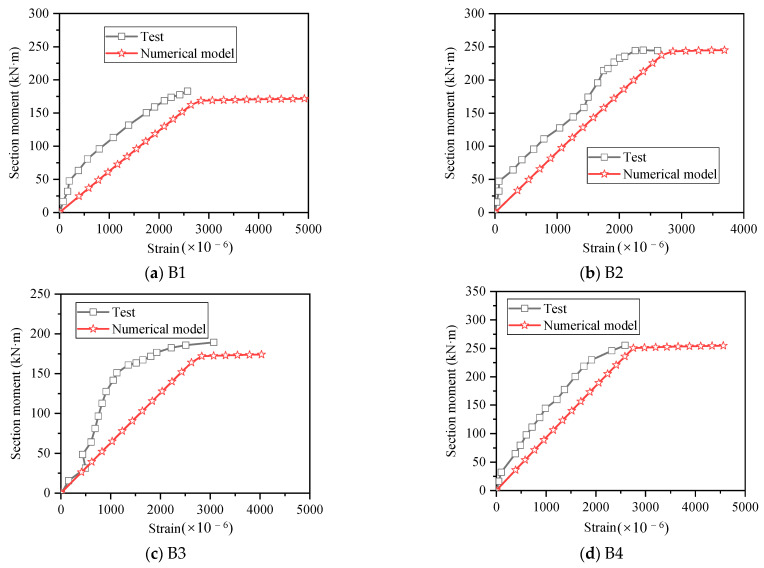
Comparison of moment–strain curves between numerical and test results.

To verify the accuracy of the numerical model, the results obtained by numerical calculation were compared with the test results. As shown in Figure 6, the moment–deflection curves obtained by the numerical model are similar to the test results, and the yield loads and ultimate loads are both consistent with the test results. Figure 7 shows the comparison of moment–strain curves of reinforcement at midspan of beam between numerical and test results, it can be found that the strain value of numerical calculation results corresponding to the same bending moment is larger than the test value, but the trend is basically the same, and this is due to the test error, and after the measured strain exceeded 2500 × 10^−6^, the strain gauges were damaged, and the measurement could not be continued. In general, the bending moment–strain curves obtained by the numerical value were reliable.

The above results proved the accuracy of the numerical model. Therefore, the numerical model and the experimental model can be considered as the test model together in the following discussion.

### 2.3. Prediction Model

The flexural capacity can be calculated using the methods introduced in the guideline GB50010-2010 [19], and the prediction model of the bearing capacity is shown as follows,

(6a) $$\alpha_{1}f_{\mathrm{ck}}{\mathrm{bx}=f}_{y}A_{s}$$

(6b) $$M\;\leq {\;\alpha }_{1}f_{\mathrm{ck}}{\mathrm{bx}(h}_{0}-\frac{x}{2})$$

where *f_y_* is the yield strength of reinforcement; *f_ck_* denotes the axis compression strength of concrete; *A_s_* is the area of reinforcement; $\;h_{0}$ is the effective section height; *x* denotes the height of compression zone;$\;\alpha_{1}$ is a constant coefficient; *M* denotes the flexural capacity of beam.

The comparison between the prediction model results and the test results in this paper is shown in Table 5, and in order to make the sample more sufficient, the results in other literature were also collected. It can be found that the calculation error of the flexural capacity of each specimen in this paper was very small, and the maximum was not more than 6%, while the results of other studies showed that the prediction model still had some errors and the maximum error was more than 20%. There are two kinds of reason for errors; one is that the strength of the reinforcement can still rise after yielding, but only the yield strength of the reinforcement was considered in the prediction model, the other is the limitation of the predictive equation and its range of applicability.

### 2.4. Model Error of Prediction Model

Generally, the test result is an objective fact; according to the above descriptions, the test results (including results from experiment and numerical model) can be approximately considered reliable. There were errors in the prediction model and the model error was an uncertain parameter. Based on this, a large number of samples were obtained through numerical calculation and combined with the experimental results to obtain the statistical information of this uncertainty. The parameter value range of the numerical model is shown in Table 6, and the parameters here are commonly used values in the design of reinforced concrete beam, including HRB500 and HRB600 HSSR, and the concrete strength grade was between C30 and C55.

**Figure 8:**
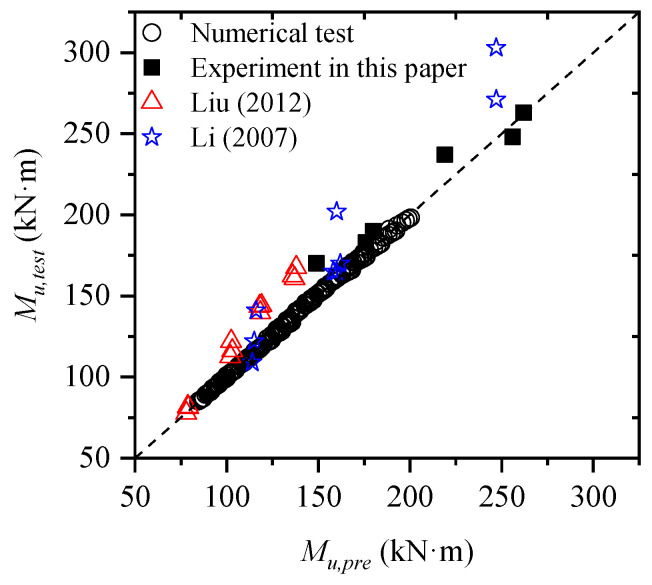
Comparison between results of prediction model and test ([24,25]).

The comparison between the test results and the prediction results is shown in Figure 8. It should be noted that the test results include the experiment results and the numerical results. On the whole, the two results are very close. To facilitate subsequent analysis, the model error μ is defined as follows,

(7) $$\mu =\frac{M_{u,test}}{M_{u,pre}}$$

where $M_{u,test}$ denotes the ultimate bending moment value of the test, including numerical test and experiment; $M_{u,pre}$ denotes the predicted ultimate bending moment value that calculated by Equations (6a) and (6b).

**Figure 9:**
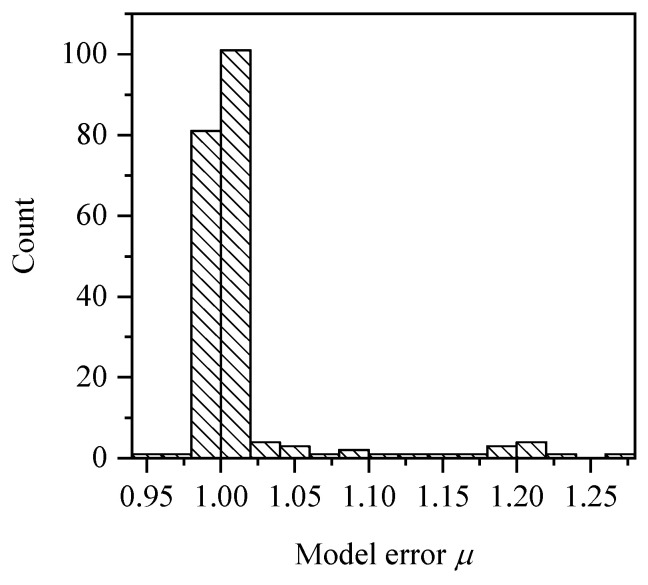
Distribution of model error *μ*.

The distribution of model error μ is shown in Figure 9. It can be seen that μ is mainly concentrated in the range of 1.0 to 1.05 and does not obey the normal distribution. The variable without explicit distribution form can be expressed by linear moment method [8,15]. The first four linear moments of model error were 1.015, 0.017, 0.0101, and 0.0087.

## 3. Reliability Analysis and Discussion

### 3.1. Limit State Function and Reliability Theory

At present, the limit state design method based on probability is main design strategy for building structure, and the basic expression of this method can be written as follows,

(8) Z=R−S

where *R* denotes the structural resistance and *S* denotes the load effect, both of them contain random variables; when *Z* > 0, the structure is at the safety state; when *Z* < 0, the structure is at the failure state; when *Z* = 0, the structure is at the limit state.

To ensure the safety and reliability of the structure, Equation (8) can be considered the following two formats, including partial safety factor format (PSSF) (Equation (9)) and resistance reduction factor format (RRFF) (Equation (10)), as shown in following,

(9) $$Z=\mu R_{n}\left(f_{ck}/\gamma_{c},\;f_{yk}/\gamma_{y}\ldots \right)-\left(\gamma_{G}S_{Gk}+\gamma_{Q}S_{Qk}\right)$$

(10) $$Z=\mu \psi R_{d}\left(f_{ck},\;f_{yk}\ldots \right)-\left(\gamma_{G}S_{Gk}+\gamma_{Q}S_{Qk}\right)$$

where $f_{ck}$ and $f_{yk}$ denote the standard value of concrete and reinforcement strength, respectively; $\gamma_{c}$ and $\gamma_{y}$ denote the partial safety factor of concrete and reinforcement, respectively; ψ denotes resistance reduction factor of bearing capacity. $S_{Gk}$ and $S_{Qk}$ denote the standard value of dead and live load, respectively; $\gamma_{G}$ and $\gamma_{Q}$ are the partial safety factor of dead and live effect, respectively, and the details value of them are shown in Table 7. Based on the limit state function, the load effect is equal to resistance, and $S_{Gk}$ and $S_{Qk}$ can be written as follows,

(11) $$\{\begin{array}{c} S_{Gk}=\frac{R_{d}}{\gamma_{G}+k\gamma_{Q}}\\ S_{Qk}=\frac{kR_{d}}{\gamma_{G}+k\gamma_{Q}} \end{array}$$

where *k* is the ratio of live load to dead load.

The reliability index *β* can be used to quantify the reliability of the structure, and there is a mapping relationship between the reliability index *β* and the failure probability of the structure. Therefore, the failure probability of Equation (9) or Equation (10) can be solved to obtain the corresponding reliability index *β*. The Monte Carlo simulation (MCS) method can be used to calculate the failure probability. In the subsequent discussion, the numbers of MCS samples *N* = 1,000,000, then the failure probability can be expressed as follows,

(12) $$P_{f}=N_{f}/N$$

where $N_{f}$ is the number of failure sample; *P_f_* denotes the failure probability.

The reliability index *β* can be obtained by the following formula,

(13) $$P_{f}=\Phi \left(\beta \right)$$

### 3.2. Statistical Characteristics of Design Parameters

According to the characteristics of HSSRCBs, eight random variables were selected, as shown in Table 8, and there were two different live loads considered for PSSF.

In order to make the calculation conditions selected for reliability calculation reasonable, some commonly used parameter ranges were selected, as shown in Table 9. The total number of cases of section considered in the design space was 4 × 4 × 8 × 4 × 2 = 1024.

### 3.3. Reliability Analysis

**Figure 10:**
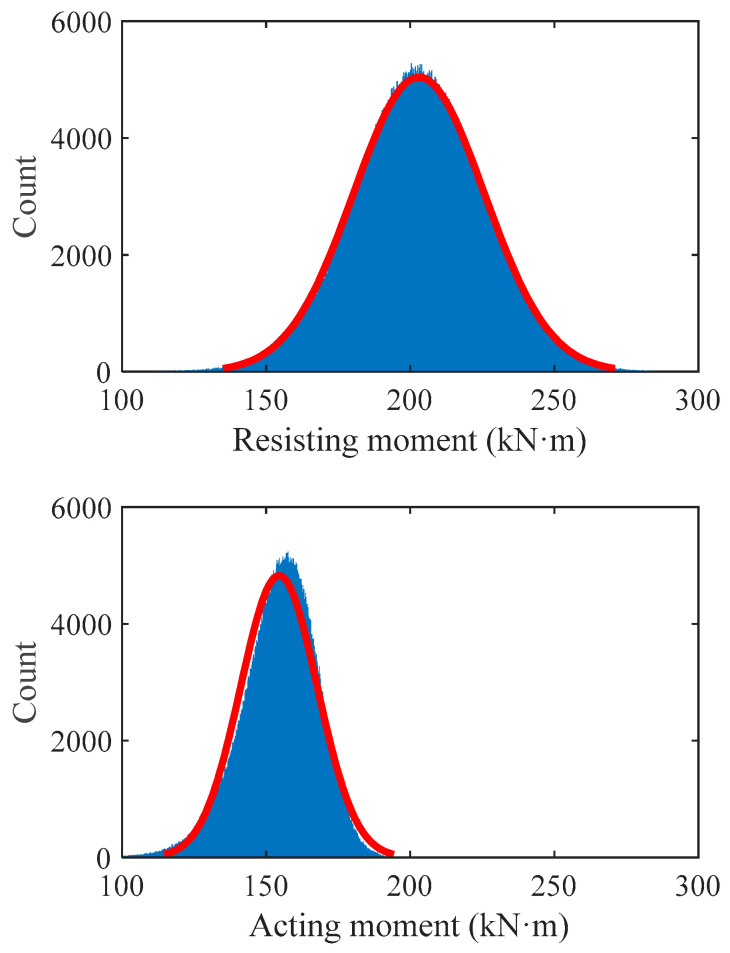
Histogram of resisting and acting moment of samples corresponding to Case I.

Table 10 shows the parameter values of case used to reflect the statistical characteristics of resistance and effect of HSSRCB. The histograms of resisting and acting moment of samples corresponding to Case I are shown in Figure 10, it can be found that when $\gamma_{s}$ = 1.0, the distribution range of resistance is wider and the value is higher than the effect, but there are still many samples whose values are less than the mean value of the load effect, which also means that the safety reliability of the case is low. Therefore, it is necessary to set material partial safety factor or resistance reduction factor.

**Figure 11:**
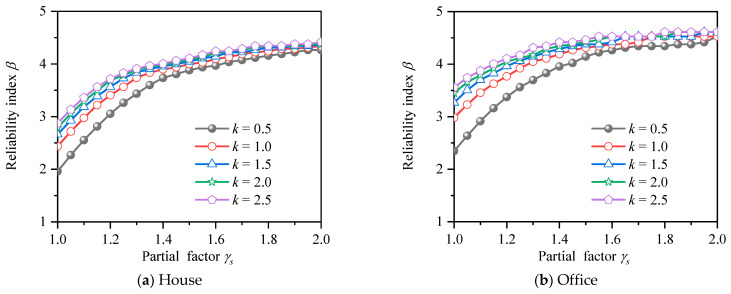
Reliability index for PSFF.

In order to analyze the influence of *k* value and *γ_s_* value on the reliability index of HSSRCB, the corresponding reliability indexes *β* of house load and office load for PSSF were calculated respectively, and the results are shown in Figure 11. It can be found that the reliability indexes *β* will increase with the increase of the partial safety factor of the material, and the increase rate will gradually decrease. When it increases to about 4.4, its value will almost not increase. Under the same material partial safety factor, the reliability index increases with the increase of *k* value. Compared with house load and office load, it can be found that the reliability index of office load is slightly larger under the same conditions.

**Figure 12:**
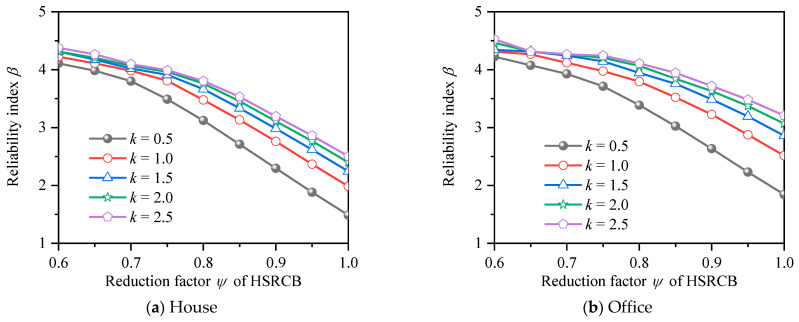
Reliability index for resistance reduction factor format.

The same method was used to calculate the reliability index under different *k* values and resistance reduction factor of HSSRCB for RRFF, and the results are shown in Figure 12. It can be seen that the reliability index decreases with the increase of the reduction resistance reduction factor, and the reduction amplitude increases with the increase of resistance reduction factor. In addition, when the reduction partial safety factor equals to 1, it means no reduction, and at this time, the reliability index of house load and office load decreases to the range of 1.4 to 2.5 and 1.8 to 3.2, respectively, which is less than the case that the PSFF does not consider the partial safety factor of materials.

### 3.4. Optimal Value of Factor

If the reliability index is too low, the structural safety cannot be guaranteed, while if the reliability index is too high, although the structural safety is increased, its economy is poor. Therefore, it is necessary to make the reliability index close to the target reliability. Due to the large number of cases (the number of cases corresponding to each material partial safety factor or resistance reduction factor is 1024 × 5 = 5120, including five *k* values, and 1024 cases, see Section 3.2), the least-square formula was used to calculate the deviation value *H* between the reliability index and the target one *β_T_*, and it can be written as,

(14) $$H=\frac{1}{n}\overset{n}{\underset{i=1}{\sum }}{\left(\beta_{i}-\beta_{T}\right)}^{2}$$

where *n* is the number of cases; $\beta_{i}$ denotes reliability index of *i*-th case, and $\beta_{T}$ denotes target reliability index, and it used the specified value of in GB 50068–2018 [29], as shown in Table 11.

#### 3.4.1. Partial Safety Factor Format (PSFF)

**Figure 13:**
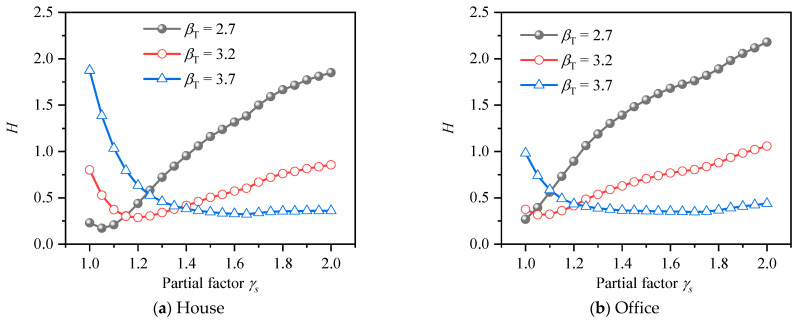
Average deviation from *β_T_* of HRB500 reinforcement for PSFF.

The average deviations from *β_T_* of HRB500 HSSR for PSFF are shown in Figure 13. It can be seen that trend of average deviation curves corresponding to different target reliability indexes is very different. Take house load as an example, when *β_T_* = 2.7 and 3.2, the average deviation value decreases first with the increase of the partial safety factor. The minimum value of the deviation value corresponding to the two kinds of target reliability is about 1.1, indicating that when the partial safety factor is about 1.1, the reliability of HSSRCB is closest to the target reliability; with the continuous increase of the partial safety factor, the deviation value becomes larger and larger, which indicates that the reliability index deviates from the target reliability, that is, although the partial safety factor meets the safety requirements, the economy is poor. When *β_T_* = 3.7, the deviation value decreases with the increase of the partial safety factor and reaches the minimum value when the partial safety factor reaches about 1.6, which is the most reasonable partial safety factor value. In the case of house load and office load, the trend of the corresponding average deviation curve is similar, but the specific values are different.

**Figure 14:**
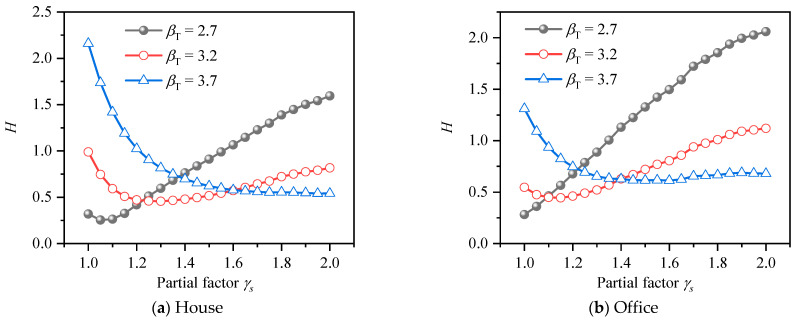
Average deviation from *β_T_* of HRB600 reinforcement for PSFF.

The average deviations from *β_T_* of HRB600 HSSR for PSFF are shown in Figure 14. Compared with HRB500 HSSR, the curve trends of house load and office load are very close.

The optimal results of partial safety factors of two kind of HSSR are summarized in Table 12. It can be seen that the strength design values of HSSR under different safety levels vary greatly. To simplify the design, it is recommended to use the strength design values corresponding to Level II, while Level I and Level III supplement the corresponding resistance reduction factor on the basis of Level II [19]. Therefore, the final partial safety factors of HSSR are shown in Table 13, the recommended strength design value of HRB500 HSSR is greater than the value specified in GB50010-2010 [19], indicating that the value in the specification is slightly conservative.

#### 3.4.2. Resistance Reduction Factor Format (RRFF)

**Figure 15:**
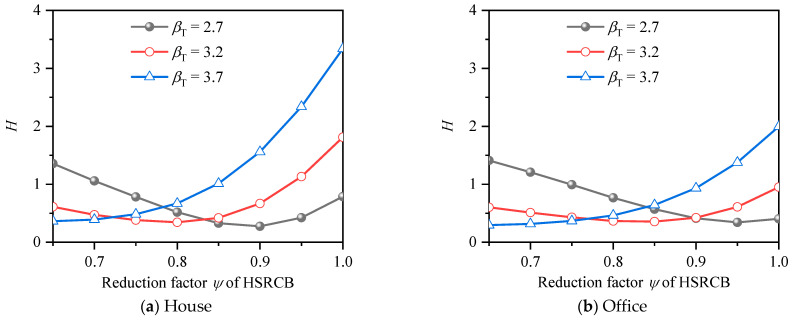
Average deviation from *β_T_* of HRB500 HSSRCB for RRFF.

**Figure 16:**
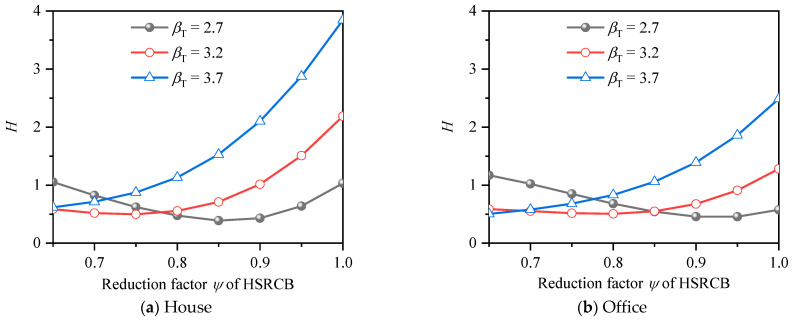
Average deviation from *β_T_* of HRB600 HSSRCB for RRFF.

The average deviations from *β_T_* for HSSRCB with HRB500 under different resistance reduction factors are shown in Figure 15. It can be found that the trends of the average deviation curves under the two conditions of house load and office load is very close, and the value of office load is obviously lower than that of house. In general, the average deviation decreases first and then increases with the growth of resistance reduction factor. The resistance reduction factor corresponding to the minimum value of the curve is the optimal value, and the larger *β_T_* is, the smaller the optimal resistance reduction factor value is, which also conforms to the rule of RRFF. Figure 16 shows the average deviation from *β_T_* for HSSRCB with HRB600, the rule is similar to that of HRB500 HSSRCB, but the specific values are different.

The optimal values of resistance reduction factor for different reinforcement grades and different safety levels are summarized in Table 14.

## 4. Conclusions

In engineering structures, improving the strength of steel reinforcement can save materials. However, the reliability of the design method of HSSR concrete structures cannot be ignored. Based on this, this paper analyzed the reliability of HSSRCBs. The flexural behaviors of HSSRCB were investigated through experiment and numerical model. The reliability of the prediction model was analyzed based on MCS. In order to obtain a suitable design method, two kinds of format for limit state function were discussed, and the average deviations from *β_T_* were calculated. Finally, the corresponding design suggestions were given, and the conclusions are as follows:

(1) The failure modes of proper HSSRCB were flexural ductility failure, and the existing bearing capacity strength design method in the specification could meet the bearing capacity calculation of HSSRCBs. Uncertainty existed in the prediction model, the discreteness of model error is not large, and model error does not obey the normal distribution.
(2) The recommended values of partial safety factor of HRB500 material were less than the specification GB50010-2010 [19]. The recommended strength design value of HRB500 was 454 MPa, which is greater than that of 435 MPa in the specification, indicating that the specification value is conservative. The recommended strength design value of HRB600 was 545 MPa, which is not given in the specification.
(3) The recommended value of bearing capacity resistance reduction factor was given, and the resistance reduction factors under different safety levels are different. Except for Level II, the resistance reduction factors of HSSRCB with HRB600 reinforcement are the same as those of HSSRCB with HRB500 reinforcement. The engineers can select the corresponding resistance reduction factor according to different structural safety levels.
(4) The investigation can provide a reference for the design of HSSRCBs. However, because only the reliabilities of flexural bearing capacity of HSSRCB were calculated, resistance reduction factor of HSSRCB and strength design value of HSSR are only applicable to the design and verification of bending bearing capacity of HSSRCBs. Reliability research on more concrete members with HSSR will be carried out in the future.

## Figures and Tables

**Table 1:** Parameter details of beam specimens.

Specimen Number	Strength Grade of Concrete	Longitudinal Reinforcement	Reinforcement Ratio (%)
B1	C50	2R25	0.982
B2	C50	3R25	1.473
B3	C70	2R25	0.982
B4	C70	3R25	1.473

Note: R denotes HRB500 grade steel reinforcement, and the number in front and behind of R represents the number and the diameter of reinforcement, respectively.

**Table 2:** Mechanical properties of HSSR.

	Diameter (mm)	Yield Strength (MPa)	Ultimate Strength (MPa)
HRB500	25	540	675
HRB400	10	446	586

**Table 3:** Mechanical properties of concrete.

	Cube Compressive Strength *f_cu,k_* (MPa)	Axial Compressive Strength *f_ck_* (MPa)
C50	53.81	34
C70	72.73	46

**Table 4:** Key points of result.

Specimen	Yield Moment (kN·m)	Yield Deflection (mm)	Ultimate Moment (kN·m)	Deflection Corresponding to Ultimate Load (mm)	Ultimate Deflection (mm)	Ductility Index	Failure Mode
B1	159	10.22	183	38	41.60	4.1	Flexural ductile failure
B2	220	11.89	248	22	26.06	2.2	Flexural ductile failure
B3	153	11.19	190	36	37.17	3.2	Flexural ductile failure
B4	247	12.62	264	16	25.28	2.0	Flexural ductile failure

Note: Ductility index is the ratio of the ultimate deformation to that of the yield deformation [21].

**Table 5:** Ultimate flexural capacity of test results and prediction results.

No.	*b* (mm)	*h* (mm)	*f_ck_* (MPa)	*f_y_* (MPa)	*ρ_s_* (%)	*M_u,exp_* (kN·m)	*M_u,pre_* (kN·m)	Error (%)	Source
B1	250	400	34	540	0.98	183	176	−3.8	Experiment in paper
B2	250	400	34	540	1.47	248	256	3.2	
B3	250	400	46	540	0.98	190	179	−5.8	
B4	250	400	46	540	1.47	264	263	−0.4	
L1	200	405	14.33	525	0.6	81.8	78.85	−3.6	Liu (2012) [24]
L2	204	400	14.33	550	0.8	122.08	102.5	−16.0	
L3	198	401	14.33	530	1.0	139.8	118.3	−15.4	
L4	201	402	14.33	505	1.3	160.76	137	−14.8	
LX1-A	200	402	14.78	525	0.6	81.21	79	−2.7	
LX2-A	199	401	14.78	550	0.8	116.33	103	−11.5	
LX3-A	202	400	14.78	530	1.0	144	119	−17.4	
LX4-A	201	403	14.78	505	1.3	167.55	138	−17.6	
LX1-B	203	402	14.05	525	0.6	77.65	78	0.5	
LX2-B	200	401	14.05	550	0.8	112.67	102	−9.5	
LX3-B	203	400	14.05	530	1.0	143.51	118	−17.8	
LX4-B	202	401	14.05	505	1.3	162.33	136	−16.2	
LW1	204	400	27.3	567	0.8	109	114	4.6	Li (2007) [25]
LW2	202	405	30	567	0.8	122	115	−5.7	
LW3	202	410	34.3	567	0.8	141	116	−17.7	
LW4	199	404	27.3	503	1.3	165	158	−4.2	
LW5	200	405	30	503	1.3	165	160	−3.0	
LW6	199	408	34.3	503	1.3	170	162	−4.7	
LW7	200	404	30	523	2.2	271	247	−8.9	
LW8	202	402	30	523	2.2	303	247	−18.5	
LW9	202	404	30	503	1.3	202	160	−20.8	

Note: *b* and *h* denote width and height of beam section; *f_c_* denotes axial compressive strength of concrete; *f_y_* denotes yield strength of reinforcement; *ρ_s_* denotes reinforcement ratio; *M_u,exp_* and *M_u,pre_* denote experimental moment and prediction moment of beam; Error = (*M_u,pre_* − *M_u,exp_*)/*M_u,exp_.*

**Table 6:** The range of parameter in the cases of numerical model.

Parameter	*b* (mm)	*h* (mm)	*f_ck_* (MPa)	*f_y_* (MPa)	*ρ_s_* (%)
Range of value	200 to 250	400 to 500	20 to 35	500 and 600	0.4 to 1.0

**Table 7:** Value of partial safety factor of load effect.

Item	Symbol	Value of PSFF	Value of RRFF
Dead load	$\gamma_{G}$	1.2	1.2
Live load	$\gamma_{Q}$	1.6	1.4

**Table 8:** Random variables.

Random Variable	Bias	COV	Distribution	Source
Section width	1	0.02	Normal	Lu et al. [26]
Section height	1	0.01	Lognormal	Lu et al. [26]
Area of steel reinforcement	1	0.03	Normal	Lu et al. [26]
Strength of concrete	1.15	0.15	Lognormal	Ribeiro and Diniz [27]
Strength of HRB500	1.08	0.075	Normal	Lu et al. [26]
Strength of HRB600	1.08	0.075	Normal	Lu et al. [26]
Dead load (Equation (5))	1.05	0.10	Normal	Galambos et al. [28]
Live load (Equation (5))	1.00	025	Extreme type I	Galambos et al. [28]
Dead load (Equation (6))	1.06	0.075	Normal	GB 50086 [29]
Live load (house) (Equation (6))	0.644	0.233	Extreme type I	GB 50086 [29]
Live load (office) (Equation (6))	0.524	0.288	Extreme type I	GB 50086 [29]

Bias = Mean value/standard value; COV denotes coefficient of variation.

**Table 9:** Design space.

Variable	Unit	Range	Interval
*b*	mm	150 to 300	50 mm
*h*	mm	400 to 550	50 mm
*ρ_s_*	%	0.5 to 2.0	0.2%
*f_c_*	MPa	16.7, 20.1, 23.4, 26.8	-
*f_y_*	MPa	500, 600	-

**Table 10:** Parameter of Case I.

	*b* (mm)	*h* (mm)	*ρ_s_* (%)	Grade of Concrete	Grade of Steel Reinforcement	*k*	$\gamma_{s}$
Case I	200	450	1.3	C30	HRB600	1.0	1.0

**Table 11:** Target reliability index.

	Level I	Level II	Level III
$\beta_{T}$	3.7	3.2	2.7

**Table 12:** Summary of optimal partial safety factor of HSSR.

Type	Standard Value of Strength (MPa)	Partial Safety Factor (Design Value of Strength)		
		Level I	Level II	Level III
HRB500	500	1.55 (322 MPa)	1.10 (454 MPa)	1.05 (476 MPa)
HRB600	600	1.70 (353 MPa)	1.10 (545 MPa)	1.05 (571 MPa)

**Table 13:** Recommendation for design strength of HSSR.

Type	Standard Value of Strength (MPa)	Recommendation for Design Strength (MPa)	Design Strength in GB50010-2010 [19] (MPa)
HRB500	500	454	435
HRB600	600	545	No value

**Table 14:** Summary of optimal resistance reduction factor of HSSRCB.

Type	Standard Value of Strength (MPa)	Resistance Reduction Factor		
		Level I	Level II	Level III
HRB500	500	0.65	0.80	0.90
HRB600	600	0.65	0.75	0.90

## Data Availability

Not applicable.

## Notations

Symbol	Meaning
$\alpha_{1}$	The constant coefficient
$f_{c}$	The compression strength of concrete
b	Width of section
x	The height of compression zone
${\;f}_{y}$	The yield strength of reinforcement
$A_{s}$	The area of reinforcement
*M*	The flexural capacity of beam
*ρ_s_*	Reinforcement ratio
*M_u,exp_*	Experimental moment
*M_u,pr_*	Prediction moment
$\sigma_{c}$	The concrete stress
$\varepsilon_{c}$	The concrete strain
$\varepsilon_{c0}$	The compressive strain when the stress just reaches *f_c_*
$\varepsilon_{\mathrm{cu}}$	The ultimate compressive strain
$A_{si}$	The area of *i*-th steel reinforcement
$\sigma_{si}$	The stress of *i*-th steel reinforcement
$y_{sj}$	The location of *i*-th reinforcement
*n_s_*	The number of steel reinforcement
ϕ	The curvature of section
*L*	The span of beam
R	The structural resistance
S	The load effect
*Z*	The limit state function
μ	Model error
$f_{ck}$	The standard value of concrete strength
$f_{yk}$	The standard value of reinforcement strength
$\gamma_{c}$	The partial safety factor of concrete
$\gamma_{y}$	The partial safety factor of reinforcement
ψ	The resistance reduction factor of bearing capacity
$S_{Gk}$	The standard value of dead load
$S_{Qk}$	The standard value of live load
$\gamma_{G}$	The partial safety factor of dead load
$\gamma_{Q}$	The partial safety factor of live load
$R_{d}$	The standard value resistance
$P_{f}$	The failure probability
$N_{f}$	The number of failure sample
*N*	The number of MCS sample
*β*	Reliability index

## References

[1] Shahrooz B.M., Reis J.M., Wells E.L., Miller R.A., Harries K.A., Russell H.G. (2014). Flexural Members with High-Strength Reinforcement: Behavior and Code Implications. J. Bridge Eng..

[2] Harries K.A., Shahrooz B.M., Soltani A. (2012). Flexural Crack Widths in Concrete Girders with High-Strength Reinforcement. J. Bridge Eng..

[3] Li Y., Aoude H. (2020). Influence of Steel Fibers on the Static and Blast Response of Beams Built with High-Strength Concrete and High-Strength Reinforcement. Eng. Struct..

[4] Liao Z., Tang D., Li Z., Xue Y., Shao L. (2019). Study on Explosion Resistance Performance Experiment and Damage Assessment Model of High-Strength Reinforcement Concrete Beams. Int. J. Impact Eng..

[5] Zhao J., Liang J., Chu L., Shen F. (2018). Experimental Study on Shear Behavior of Steel Fiber Reinforced Concrete Beams with High-Strength Reinforcement. Materials.

[6] Deng Z., Huang S., Wang Y., Xue H. (2022). Experimental Research on Fatigue Behavior of Prestressed Ultra-High Performance Concrete Beams with High-Strength Steel Bars. Structures.

[7] Zhang P., Xu F., Liu Y., Ahmed Sheikh S. (2022). Shear Behaviour of Composite Beams with Permanent UHPC Formwork and High-Strength Steel Rebar. Constr. Build. Mater..

[8] Zhao Y.-G., Tong M.-N., Lu Z.-H., Xu J. (2020). Monotonic Expression of Polynomial Normal Transformation Based on the First Four L-Moments. J. Eng. Mech..

[9] Tong M.-N., Zhao Y.-G., Lu Z.-H. (2021). Normal Transformation for Correlated Random Variables Based on L-Moments and Its Application in Reliability Engineering. Reliab. Eng. Syst. Saf..

[10] Zhang X.-Y., Lu Z.-H., Wu S.-Y., Zhao Y.-G. (2021). An Efficient Method for Time-Variant Reliability Including Finite Element Analysis. Reliab. Eng. Syst. Saf..

[11] Chen G., Yang D. (2019). Direct Probability Integral Method for Stochastic Response Analysis of Static and Dynamic Structural Systems. Comput. Methods Appl. Mech. Eng..

[12] Li X., Chen G., Cui H., Yang D. (2021). Direct Probability Integral Method for Static and Dynamic Reliability Analysis of Structures with Complicated Performance Functions. Comput. Methods Appl. Mech. Eng..

[13] Chen G., Yang D. (2021). A Unified Analysis Framework of Static and Dynamic Structural Reliabilities Based on Direct Probability Integral Method. Mech. Syst. Signal Process..

[14] Zhou Y., Zhang J., Li W., Hu B., Huang X. (2020). Reliability-Based Design Analysis of FRP Shear Strengthened Reinforced Concrete Beams Considering Different FRP Configurations. Compos. Struct..

[15] Zhang W., Liu X., Huang Y., Tong M.-N. (2022). Reliability-Based Analysis of the Flexural Strength of Concrete Beams Reinforced with Hybrid BFRP and Steel Rebars. Archiv. Civ. Mech. Eng..

[16] Wakjira T.G., Ibrahim M., Ebead U., Alam M.S. (2022). Explainable Machine Learning Model and Reliability Analysis for Flexural Capacity Prediction of RC Beams Strengthened in Flexure with FRCM. Eng. Struct..

[17] Wakjira T.G., Ebead U., Alam M.S. (2022). Machine Learning-Based Shear Capacity Prediction and Reliability Analysis of Shear-Critical RC Beams Strengthened with Inorganic Composites. Case Stud. Constr. Mater..

[18] Shen L., Shen Y., Liang S. (2022). Reliability Analysis of RC Slab-Column Joints under Punching Shear Load Using a Machine Learning-Based Surrogate Model. Buildings.

[19] (2010). Code for Design of Concrete Structures. China Academy of Building Research.

[20] Liu Z., Zeng Z., Liu X., Zhao R. (2020). Analysis of Flexural Ductility of Reinforced Concrete Beams with 500 MPa Steel. J. Huaqiao Univ. (Nat. Sci.).

[21] El-Sherif H., Wakjira T.G., Ebead U. (2020). Flexural Strengthening of Reinforced Concrete Beams Using Hybrid Near-Surface Embedded/Externally Bonded Fabric-Reinforced Cementitious Matrix. Constr. Build. Mater..

[22] Melo G.F., de Torii A.J., Medeiros E.M., de Kzam A.K.L. (2021). MATLAB Computational Routines for Moment-Curvature Relation of Reinforced Concrete Cross Sections. Rev. IBRACON Estrut. Mater..

[23] Fujikake K., Li B., Soeun S. (2009). Impact Response of Reinforced Concrete Beam and Its Analytical Evaluation. J. Struct. Eng..

[24] Liu P. (2012). Experimental and Theoretical Research on Beams with HRB500 High Strength Steel Bars.

[25] Li Y. (2007). Experimental Research on Behaviors of Reinforced Concrete Beams with 500 mpa Steel Bars.

[26] Lu R., Luo Y., Conte J.P. (1994). Reliability Evaluation of Reinforced Concrete Beams. Struct. Saf..

[27] Ribeiro S.E.C., Diniz S.M.C. (2013). Reliability-Based Design Recommendations for FRP-Reinforced Concrete Beams. Eng. Struct..

[28] Galambos T., Ellingwood B., MacGregor J., Cornell C. (1982). Probability Based Load Criteria: Assessment of Current Design Practice. J. Struct. Div..

[29] (2019). Unified Standard for Reliability Design of Building Structures.

